# Antibiotic Hypersensitivity Mechanisms

**DOI:** 10.3390/pharmacy7030122

**Published:** 2019-08-27

**Authors:** Jenana H. Maker, Cassandra M. Stroup, Vanthida Huang, Stephanie F. James

**Affiliations:** 1 Department of Pharmacy Practice, Thomas J Long School of Pharmacy and Health Sciences, University of the Pacific, Stockton, CA 95211, USA; 2 Department of Pharmacy Practice, School of Pharmacy, Rueckert-Hartman College of Health Professions, Regis University, Denver, CO 80221, USA; 3 Department of Pharmacy Practice, College of Pharmacy-Glendale, Midwestern University, Glendale, AZ 85308, USA; 4 Department of Pharmaceutical Sciences, School of Pharmacy, Rueckert-Hartman College of Health Professions, Regis University, Denver, CO 80221, USA

**Keywords:** antimicrobial, hypersensitivity, antibiotic, allergy, anaphylaxis, Type I, Type II, Type III, Type IV

## Abstract

Antibiotics are commonly prescribed to treat a variety of bacterial infections. As with all medications, hypersensitivity reactions may occur and clinicians should be able to recognize them accurately and recommend appropriate management. Antibiotic related hypersensitivity reactions may be one of four different types: Type I reactions, which are IgE mediated and may lead to anaphylaxis; Type II reactions that are antibody-mediated and may result in thrombocytopenia, neutropenia, or hemolytic anemia; Type III reaction that involves an immune complex formation such as vasculitis; and Type IV reactions that consist of four subtypes and typically include a rash of varying level of severity with or without systemic signs and symptoms. Herein, we describe the mechanisms of different types of allergic reactions to commonly prescribed antibiotics and offer recommendations for management. Further, we briefly refer to antibiotic reactions that mimic hypersensitivity reactions but are not immune mediated, such as pseudoallergies and serum sickness-like reactions.

## 1. Introduction

The discovery of antibiotics to treat bacterial infections has been one of the greatest achievements in medicine. Such drugs may be used to treat a variety of diseases, from mild to life threatening. However, drug hypersensitivity reactions (DHR) can exclude some patients from receiving these medications. Patients with true DHR need to be promptly identified and managed as they may develop life-threatening complications from the offending drug. Understanding the underlying immunologic mechanisms of DHR can help clinicians distinguish between true hypersensitivity vs. inaccurate and potentially harmful antibiotic allergy diagnoses. If DHR is suspected, further work-up into classifying the immune-mediated process will be necessary to appropriately manage the patient both acutely and long-term.

Different classification systems have been developed to categorize DHR. The International Consensus on drug allergy (ICON) classifies DHR clinically by the onset of actions as either immediate or delayed [1]. Immediate reactions are usually IgE-mediated and occur within the first hour of drug administration, while delayed reactions are a heterogenous group of immune mediated reactions that typically take days to weeks to occur. The Gell and Coombs system classifies DHR by their immunologic mechanism into four categories (Type I–IV) [2]. Type I reactions are immediate and IgE-mediated, where IgE antibodies specific to the allergen bind to mast cells and trigger the release of mediators such as histamine and leukotrienes, among others, to cause vasodilation and increased capillary permeability [1,2,3,4,5,6]. Type II, III, and IV reactions are delayed in onset. Specifically, type II hypersensitivities are the result of antibodies binding to cell surface antigens, leading to an antibody-dependent cell-mediated cytotoxicity. Type III hypersensitivities are due to the development of an immune complex formation which deposits in vasculature and tissues, activating the complement that releases inflammatory mediators [1,2,3,4,5,6]. Lastly, type IV hypersensitivity is mediated by T cells and occurs when T cells are sensitized to an antigen. These antigens may be the drug itself or its metabolite. Such products may then bind to serum or cell bound proteins, creating an immunogenic molecule, which is subject to the antigen processing pathway and presentation to T cells. Type IV reactions are further subcategorized into four groups (IVa, IVb, IVc, IVd) depending on the specific T cell type involved [1,2,3,4,5,6,7].

Hereby, we review the various types of antibiotic hypersensitivity, clinical presentation, diagnosis, and treatment plans. A general description and common features of four hypersensitivity types are summarized in Table 1.

## 2. Type I Reactions

Anaphylaxis is a severe and potentially life-threatening hypersensitivity reaction that typically involves multiple organ systems. The incidence of anaphylaxis is estimated to range between 3 to 50 per 100,000 person-years and a lifetime prevalence of less than 2% [8]. Antibiotics are one of the leading causes of anaphylaxis with beta-lactams being most commonly implicated. Broadly speaking, anaphylaxis may be IgE-dependent, IgE-independent, or non-immunologic.

### 2.1. Immune-Mediated IgE-Dependent Anaphylaxis

The IgE-mediated reactions occur when an allergen-specific IgE binds to Fc-epsilon-RI IgE receptors on mast cells and basophils, leading to mast cell degranulation and release of multiple mediators, enzymes, and cytokines that trigger typical signs and symptoms of anaphylaxis [9]. The most relevant mediators are further described below and their effects on the organ system and associated symptoms are summarized in Table 2.

#### 2.1.1. Histamine and Tryptase

Both histamine and tryptase are preformed mediators stored in the secretory granules of mast cells and released by mast cell degranulation and basophils. Histamine can bind to four types of histamine receptors (H1 through H4). The H1 and H2 receptors mediate several systemic effects of anaphylaxis including bronchoconstriction, tachycardia, hypotension, and flushing. Both H1 and H2 antagonists are used as adjunctive therapies in the treatment of anaphylaxis (further described in the Diagnosis and Treatment section). H3 and H4 receptors have not been as extensively studied but H4 receptors appear to be involved in chemotaxis and pruritus development. Histamine plasma levels correlate with the severity of anaphylaxis. However, they are typically not measured in a clinical setting as they return to baseline within 30 min of the onset of symptoms due to rapid metabolism [9,10]. Tryptase is a protease that is largely produced by the mast cells. Tryptase causes activation of the coagulation pathways and kallikrein-kinin contact system, thereby contributing to vasodilatation, hypotension, and angioedema. Since tryptase is more stable than histamine, it has been utilized as a biomarker of mast cell activation and may support the diagnosis of anaphylaxis [8,9,10,11,12].

#### 2.1.2. Platelet Activating Factor

Platelet activating factor (PAF) is produced and released by a variety of cells, including mast cells, basophils, neutrophils, eosinophils, and platelets. In addition, many of these cells can also be directly simulated by PAF. It has a short half-life of around 3 to 13 min and is inactivated by PAF acetylhydrolase (PAF-AH) [9,10]. While the role of PAF has not been as extensively studied as histamine in anaphylaxis, it appears to play a principal part in inflammation and coagulation. In the lungs, PAF increases bronchial epithelial inflammation, bronchoconstriction, and bronchial hyper-reactivity. Further, it increases vascular permeability, reduces coronary blood, and has negative intropic and arrhythmogenic effects on the cardiac tissue [13,14]. It is likely that PAF also contributes to urticaria as subcutaneous injections in volunteers induce urticarial wheals and erythema [13]. Other studies have found that PAF levels increase in proportion to the severity of anaphylaxis. At the same time, patients with anaphylaxis have significantly lower levels of PAF-AH [15]. Overall, these findings indicate that PAF is a likely contributor to the development and pathophysiologic changes in anaphylaxis.

#### 2.1.3. Other Mediators

Cysteinyl leukotrienes (CySLT) are generated from arachidonic acid via the 5-lipoxygenase pathway and are released during mast cells and basophil activation. While they have been largely studied in patients with asthma and allergic rhinitis, they are known to have multiple immunologic functions and may well be contributing to anaphylactic reactions. Studies in healthy volunteers demonstrated that subcutaneous injections of leukotriene (LT) B4, LTC4, and LTD4 induced erythema and wheal formation, while inhalational administration of LTC4 and D4 caused bronchoconstriction [16,17]. In addition to the CysLTs, mast cells release a variety of other substances including chymase, heparin, carboxypeptidase A3, and prostaglandin D2. Furthermore, multiple cytokines such as IL-4, IL-5, IL-6, interferon (IFN)-γ, and tumor necrosis factor (TNF)-α are involved and may affect cellular responsiveness to anaphylaxis [9,10]. The specific pathways and cell functions are complex and still require further study as it pertains to anaphylaxis.

### 2.2. Immune-Mediated IgE-Independent Anaphylaxis

As studies have found that patients with low to undetectable IgE levels may experience anaphylaxis, recent research has focused on identifying other immune-mediated pathways that can contribute to the development of anaphylaxis independent of IgE. It is not exactly clear how some of these processes take place, but animal studies point towards an IgG-dependent response which activates macrophages and neutrophils and leads to the release of PAF which, in turn, can also activate mast cells. Complement-mediated anaphylaxis involving production of anaphylatoxins C3a, C4a, and C5a has also been described. The IgE-independent immune-mediated reactions have been poorly studied with antibiotics so it is unclear what role, if any, they play in antibiotic-related hypersensitivity reactions [9,10,11,12,13].

### 2.3. Non-Immunologic Anaphylaxis

Some antibiotics can induce anaphylaxis via non-immunologic mechanisms. These reactions are not considered to be true DHR and are sometimes referred to as anaphylactoid or pseudoallergies, as they clinically resemble true anaphylaxis. Red man syndrome is one such example which may develop when vancomycin is rapidly infused (1 g or higher doses infused over less than 60 min) and can lead to such symptoms as flushing, redness, and pruritus affecting the upper part of the body such as face and neck. Severe symptoms such as shortness of breath or hypotension are rare but can occur as well. The underlying mechanism of this reaction is direct stimulation of mast cell degranulation by vancomycin and does not involve an immune mediated process. Patients are typically managed by reducing the rate of vancomycin infusion and premedication with antihistamines [18,19]. Quinolones is another class of antibiotics that have been shown to directly stimulate mast cell degranulation but this is a relatively rare adverse effect [20].

### 2.4. Diagnosis and Treatment

The diagnosis of anaphylaxis is largely based on the clinical presentation summarized in Table 3. Cutaneous symptoms are most common, affecting about 84% of patients [21]. Many patients also experience cardiovascular (72%) and respiratory (68%) symptoms. Symptoms develop very rapidly, with a reported median time of 5 min if the offending drug is administered parenterally [21].

**Figure 1:**
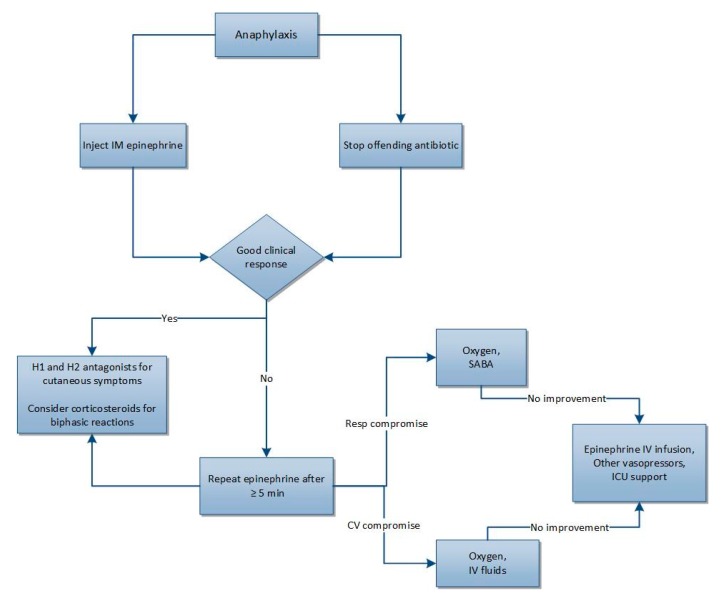
Treatment overview of anaphylaxis [21,22]. CV: cardiovascular. ICU: intensive care unit. IM: intramuscular. IV: intravenous. Resp: respiratory. SABA: short-acting beta2 agonist.

Intramuscular epinephrine is considered first-line therapy and should be administered as soon as possible. Further management depends on specific symptoms with which the patient is presenting, and is further outlined in Figure 1. Short-acting beta-2 agonists and antihistamines are considered second-line interventions to alleviate bronchoconstriction and cutaneous symptoms, respectively. Corticosteroids are frequently administered to prevent biphasic reactions, but evidence on their benefit is limited [21,22,23].

### 2.5. Beta-Lactam Cross-Reactivity

Beta-lactam antibiotics includes two major (penicillins and cephalosporins) and four minor (carbapenems, monobactams, oxacephems, and beta-lactamase inhibitors) classes of antibiotics [24]. The widespread use of these agents sparked debate over the risk of allergic cross-reactivity amongst these antibiotics in patients with features of an IgE-mediated reaction. While early retrospective studies indicated cross-reactivity of penicillins to cephalosporins to be upward of 40%, more recent studies have found that the actual penicillin cross-reactivity with cephalosporins and carbapenems is less than 5% and less than 1%, respectively [25]. Furthermore, patients with a history of penicillin allergy may tolerate penicillins later in life, as approximately 50% of patients lose sensitivity over 5 years and this population increases to 80% over 10 years [26]. The mechanism for which patients may lose sensitivity over time is still up for debate. Inability for prescribers to utilize the appropriate medications due to self-reported patient allergies or concern for cross-reactivity causes increased risk of broad-spectrum antibiotic use, avoidance of first-line therapies, increased health-care costs, and increased risk of antibiotic resistance [3,24,25,26,27,28]. Several relevant reviews within this special issue have been published that address beta-lactam cross-reactivity mechanisms and management in greater detail [29,30,31].

## 3. Type II Reactions

Type II reactions are delayed immune-mediated reactions that typically involve antibody-mediated cell destruction of circulating white blood cells (WBC), red blood cells (RBC), or platelets. The pathogenesis has not been fully elucidated, and different types of pathologic mechanisms have been associated with different drug classes [32]. In the case of antibiotics, the immune mediated toxicity is thought to be most likely due to hapten-dependent antibodies. Haptens are small molecules (less than 5000 Daltons) that do not elicit an immune response on their own but can become immunogenic when they covalently bind to the cell membrane of larger proteins. This leads to the production of IgG (or, less commonly IgM) antibodies that target either the bound drug or a cell membrane component altered by the drug. The subsequent interaction between the antibody and antigen destroys the cell, either via complement or phagocytosis. For example, penicillin’s beta-lactam ring can bind to the proteins on RBCs and lead to production of antibodies. There is also evidence that reactive metabolites of a drug can bind to the cell membrane and lead to the production of antibodies. This bioactivation typically occurs in the liver by the cytochrome P450 enzymes. However, neutrophil enzyme myeloperoxidase can also produce reactive drug metabolites via oxidation [33]. For example, biotransformation of SMX/TMP by the liver leads to the production of SMX-nitroso, which is highly chemically reactive and can bind to cell membrane glycoproteins [34]. The specific clinical presentation varies depending on the cell type involved but since antibodies typically target either WBC, RBC, or platelets, patients will present with neutropenia, hemolytic anemia, or thrombocytopenia, respectively.

While the exact incidence of antibiotic induced cytopenias is unknown, the overall incidence is estimated to be around a dozen cases per million population per year. Drug-induced neutropenia appears to be most common with an incidence of 3 to 16 cases, followed by thrombocytopenia (10 cases), and lastly hemolytic anemia (1 case) [35]. Since Type II reactions are rare, establishing the diagnosis can be challenging and often means excluding other causes of decreased blood counts first. Examples of differential diagnoses include sepsis, autoimmune diseases, hereditary syndromes, myelodysplasia, infections, or nutritional deficiencies. Even if a drug-related cause is suspected, many patients are taking multiple drugs and identifying the offending drug can be difficult. A thorough clinical history should be obtained that consists of a medical chart review as well as patient interview to find out which medications the patient was taking and if a temporal relationship between the suspected antibiotic ingestion and development of cytopenia can be established. To help determine the likelihood of drug-induced causes, a clinical scoring system has been developed for drug-induced thrombocytopenia (Table 4), but may potentially serve as an aid to identify drug-induced neutropenia and anemia as well [36].

### 3.1. Thrombocytopenia

Most cases of drug-induced thrombocytopenia develop after 5 to 10 days of the exposure to the antibiotic. Patients typically present with signs and symptoms of bleeding such as petechiae, bruising, and epistaxis. In case of severe thrombocytopenia (platelet count <20,000 cells/µL), life-threatening hemorrhage can occur. Antibiotics most commonly implicated in thrombocytopenia include penicillin, ceftriaxone, SMX/TMP, vancomycin, and rifampin. Once the offending antibiotic has been discontinued, the recovery of platelet counts is typically observed within about one week. Since drug-dependent platelet antibodies can persist for years, patients should be counseled to avoid any future exposure, as thrombocytopenia can develop within hours of repeat exposure. In addition to drug discontinuation, patients with severe thrombocytopenia or hemorrhage may require platelet transfusions [32,36,37].

### 3.2. Neutropenia

Neutropenia can be mild (absolute neutrophil count (ANC), <1–1.5 × 10^9^ cells/L), moderate (ANC 0.5–1 × 10^9^ cells/L) or severe (ANC <0.5 × 10^9^ cells/L) [38]. While the specific clinical presentation depends on the degree and duration of neutropenia, most patients often present with fevers, chills, and myalgias. The symptoms may occur in conjunction with a systemic infection such as pneumonia and may progress to sepsis if left untreated [39]. Antibiotics with well-established associations with neutropenia include penicillins, cephalosporins, vancomycin, SMX/TMP, dapsone, chloramphenicol, and macrolides.

Similar to drug-induced thrombocytopenia, discontinuation of the offending agent is the mainstay treatment. Neutrophil counts take, on average, eight days to recover (range 2–20 days), but the recovery time can be longer in those with severe neutropenia and/or septic complications [40]. Patients with a suspected infection or sepsis may need to be treated with broad-spectrum antibiotics. Granulocyte colony stimulating factors (G-CSF) such as filgrastim may be appropriate in patients with poor prognostic factors (presence of sepsis, age over 65 years, severe neutropenia, or multiple comorbidities), as it may hasten the recovery of WBCs and shorten the duration of hospitalization and antibiotic therapy [40,41].

### 3.3. Hemolytic Anemia

Antibiotic-induced hemolytic anemia is most commonly associated with cefotetan, followed by ceftriaxone, piperacillin, and penicillin. Patients presenting with acute anemia will typically have a history of receiving the antibiotic before, without any notable adverse effects. However, when the drug is introduced for the second time in the presence of drug-induced antibodies, a hemolytic event may occur. The exception to this is cefotetan induced anemia, which may occur days after a single dose in patients who have never received cefotetan before (e.g., prophylactic use during surgery). It has been proposed that the immune system may be primed by meat ingestion originating from farm animals who have been given antibiotics. This can lead to the production of weak cefotetan antibodies and a subsequent hemolytic reaction after only one cefotetan administration [42].

In patients with suspected antibiotic-induced hemolytic anemia, treatment mainly consists of immediate discontinuation of the offending drug. Anemia typically resolves shortly after antibiotic discontinuation. Again, cefotetan may be an exception as cefotetan-bound RBCs could be found for up to 98 days after receiving the drug, indicating that the drug remains bound for the duration of the RBC life span [43]. Patients with severe anemia may additionally require RBC transfusions.

## 4. Type III Reactions

Type III reactions occur when IgG or IgM antibodies form immune complexes with drugs [1,3,7,44]. Normally, these complexes are promptly removed; however, occasionally they can precipitate and activate the complement pathway. The complement components recruit and activate neutrophils and macrophages, which results in tissue inflammation and injury. The site of the antigen–antibody complex deposition and not the source of the antigen is what determines the cluster of symptoms. As a result, the complexes deposited in blood vessels, kidneys, and joints, will present as vasculitis, nephritis, and arthritis, respectively [45]. The reaction time can take anywhere from days to weeks to develop. Examples of Type III reactions include serum sickness and small vessel vasculitis [1,3,7].

Serum sickness is typically characterized by fever, urticarial rash, and polyarthralgia. True serum sickness has largely been implicated with murine or chimeric monoclonal antibodies [46]. However, several antibiotics may mimic serum sickness and cause serum sickness-like reactions (SSLR). Symptoms are similar albeit milder than classic serum sickness, but there is no formation of immune complexes or complement activation. Antibiotics commonly implicated with this reaction include cephalosporins (particularly cefaclor), penicillins, sulfonamides, and ciprofloxacin [47,48]. The exact mechanism has not been fully elucidated, but it is thought that the drugs either act as haptens or produce metabolites that are toxic to the cells. Removal of the offending antibiotic is usually sufficient to treat this reaction and resolve the symptoms [49].

The immune complex deposition in the small vessels can lead to the development of small vessel vasculitis, which is usually limited to the cutaneous tissue and does not involve additional organs. In many cases of antibiotic induced vasculitis, the inflammatory mediator infiltrating the cells are the neutrophils, which is then referred to as leukocytoclastic vasculitis. Patients typically present with purpura and/or petechiae, and urticarial lesions may also be present [50]. Antibiotics most commonly implicated with this reaction include penicillins and cephalosporins [51,52]. Treatment typically consists of discontinuing the offending antibiotic and, in more severe cases, corticosteroid therapy.

## 5. Type IV Reactions

### 5.1. Type IVa

Type IVa is described as a type 1 helper (Th-1) lymphocyte mediated response leading to macrophage activation by interferon (IFN)-γ secretion [4,34]. Allergic contact dermatitis induced by topical antibiotics is an example of such a reaction, and consists of a sensitization and elicitation phase [53]. The sensitization phase is initiated by hapten binding to skin carrier proteins. This hapten–protein complex is engulfed and processed by the antigen presenting cells (such as the dermal dendritic cells) which migrate to the regional lymph nodes and prime the T cells. The sensitization period typically lasts between 10 to 15 days. If the individual is exposed to the same hapten again, the elicitation phase occurs within 24 to 72 h. In this phase, the activated CD8+ T cells, the main effector cells associated with allergic contact dermatitis, are recruited to the skin where they induce keratinocyte apoptosis and release inflammatory cytokines such as IFN-γ and TNF-α, among others [53]. The clinical manifestations include erythema, pruritus, and formation of scaly plaques or vesicles. The most commonly implicated antibiotics are topical agents such as bacitracin, polymyxin B, and neomycin [54]. If medication history and timing are uncertain, patch testing can be implemented, as it is considered gold standard for diagnosing allergic contact dermatitis [55]. Prompt discontinuation and avoidance of further exposure of the offending drug are the mainstays of therapy. Topical corticosteroids or calcineurin inhibitors may be considered for a more rapid control of symptoms.

### 5.2. Type IVb

Type IVb reactions are mediated by a Th-2 type immune response and result in eosinophilic inflammation, including the release of IL-4 and IL-5 cytokines [4,34]. One clinical syndrome associated with this reaction is Drug Reaction with Eosinophilia and Systemic Symptoms (DRESS), which is a serious and potentially life-threatening drug hypersensitivity reaction with mortality rates estimated to be close to 10% [56]. The pathogenesis has not been fully elucidated and different causes have been identified including drugs (most commonly antibiotics and anticonvulsants) and reactivation of human herpes viruses, particularly HHV-6. More recent evidence suggests that a dysregulation of regulator T cells contributes to the development of this syndrome [57]. Further, genetic susceptibility may be strongly associated with the risk of developing DRESS with certain drugs if they preferentially bind to specific HLA alleles. In this case, drugs are able to stimulate T cells directly by binding directly to the T cell receptors or major histocompatibility complex (MHC)/human leukocyte antigen (HLA) [58]. One of the best known examples in infectious diseases is the association between abacavir hypersensitivity and HLA*B57:01 allele. The presence of HLA*B57:01 allele increases the risk of a hypersensitivity reaction to around 50%, and the HIV guidelines recommend HLA testing before abacavir therapy is initiated. If a patient tests positive, abacavir should be documented in the patient’s allergy list within the medical health record [59]. Other examples include dapsone hypersensitivity with HLA*B13:01 allele and flucloxacillin-induced hepatotoxicity with HLA-B*57:01 [58,59,60].

The incidence of antibiotic induced cases of DRESS is unknown, but is likely variable depending on the specific drug and the immune status of the individual. Amongst antibiotics, the most commonly implicated drugs are SMX-TMP, vancomycin, minocycline, and less frequently, beta lactam antibiotics [56,57,58,59,60]. The typical clinical manifestations of DRESS include a maculopapular rash, eosinophilia and/or atypical lymphocytes, systemic symptoms including lymphadenopathy, and organ involvement (liver, kidneys, lung, or heart) [61]. The onset of this reaction is delayed and ranges between 2 to 8 weeks [56].

The prognosis of DRESS is variable and may not resolve shortly after the offending drug has been discontinued. Some patients may continue to experience symptoms weeks to months afterwards. For mild cases, moist dressing, emollients and topical corticosteroids may provide symptomatic relief. Systemic corticosteroids are typically administered to patients with severe organ involvement despite lack of conclusive evidence of their benefit [56]. Recent case reports indicate that a short course of cyclosporine may hasten recovery of organ function and resolution of rash [62].

### 5.3. Type IVc

Type IVc reactions are mediated by cytotoxic CD8+ T cells that induce apoptosis or necrosis of the keratinocytes [3,34]. These reactions manifest as bullous skin reactions, such as Steven–Johnson Syndrome (SJS) and Toxic Epidermal Necrolysis (TEN). SJS and TEN are a spectrum of diseases that are distinguished based on the percentage of body surface that is with epidermal detachment, as described in Table 5. SJS/TEN is a rare hypersensitivity reaction that has an estimated incidence of about 6 cases per million persons per year [63]. However, certain patients appear to be at an increased risk, particularly those with HIV, malignancy, and certain HLA alleles [63,64,65].

The pathogenesis of SJS/TEN is largely driven by cytotoxic CD8+ T cells and natural killer (NK) cells which induce keratinocyte apoptosis and necrosis. Other cytotoxic proteins that are released in this process include granulysin, perforin/granzyme, Fas ligand, TNF-α, and IFN-γ [66,67]. The hallmark finding associated with SJS/TEN is epidermal necrosis. The skin is erythematous with blister formation, atypical target lesions, or bullae, and may detach when rubbed gently (positive Nikolsky sign). Mucosal involvement and systemic symptoms (fever, pain, myalgias, headache, etc.) are also common [66,67]. SJS/TEN carry mortality rates between 10 to 30% since an extensive loss of skin leads to fluid and electrolyte loss, sepsis, superinfection, and multiorgan failure [66,67,68]. The most commonly associated antibiotics are SMX/TMP, nevirapine, quinolones, and beta lactams with a median time of onset of about 4 weeks [66,67].

While there is no standardized management approach, aggressive supportive care and a multidisciplinary approach are important interventions that aim to decrease mortality and improve outcomes. Patients typically need to be referred to burn intensive care units for appropriate wound care. Additional specialties such as ophthalmology, urology, or dermatology may need to be consulted depending on specific patient needs. In addition, fluid, electrolyte, and nutrition replacement, pain control, and antibiotics for prevention of superinfection may be required [66]. Treatment with systemic corticosteroids and intravenous immunoglobulin (IVIG) is controversial, as studies have not consistently demonstrated benefit [66,69]. Newer evidence has demonstrated possible survival benefit with use of cyclosporine and TNF inhibitors, but further studies are needed as currentdata largely come from small open-label case series [66,67].

### 5.4. Type IVd

Type IVd reactions result from neutrophilic inflammation via T lymphocytes [3,34]. Antibiotic induced causes of this reaction typically manifest as acute generalized exanthematous pustulosis (AGEP), which is a rare hypersensitivity reaction with an incidence of 1 to 5 cases per million per year. The clinical manifestations of AGEP include fever, erythema, and development of numerous small sterile pustules, typically within 24 to 48 h of drug exposure. Organ involvement is uncommon and has been reported in less than 20% of cases, most commonly, liver, and less frequently, kidneys and lungs [70,71].

The pathogenesis of AGEP involves activation of CD4+ and CD8+ T cells that migrate to the skin and induce keratinocyte apoptosis. In addition, these cells release CXCL8 that stimulates neutrophil chemotaxis resulting in sterile pustule formation. Other chemokines such as IFN-γ and macrophage colony stimulating factor also stimulate CXCL8 release by keratinocytes and further propagate neutrophil accumulation [70]. The most commonly implicated antibiotics are aminopenicillins, quinolones, SMX/TMP, and some antifungals (terbinafine, fluconazole). The prognosis of AGEP is generally favorable and lesions typically resolve within 2 weeks after drug discontinuation. Supportive therapies such as moist dressings and antiseptic solutions may help prevent superinfection, while topical corticosteroids can be considered for symptomatic relief [70,71].

## 6. Conclusions

Drug hypersensitivity reactions related to antibiotics are broadly classified into four groups, based on the underlying immunologic process. Each type has diverse manifestations, onset, and severity of disease. The risk of a hypersensitivity reaction varies widely between different antibiotics and some drug classes such as beta-lactams, can cause both immediate and delayed reactions. Treatment typically consists of discontinuation of the offending drug but, depending on the severity of the reaction, additional supportive measures may be necessary.

## Figures and Tables

**Table 1:** Summary of immune-mediated antibiotic hypersensitivity reactions [1,2,3,4,5,6,7].

Type	Description	Pathogenesis	Onset of Reaction	Typical Clinical Findings	Commonly Associated Antibiotics
I (Immediate)	IgE-mediated hypersensitivity	Antibiotic-specific IgE binds to Fc-epsilon-RI receptors on mast cells and basophils. Subsequent antibiotic exposure leads to mast cell and basophil degranulation	<1 h	Anaphylaxis, hives, angioedema, N/V, abdominal pain, SOB, wheezing, anxiety, confusion, chest pain, palpitations, syncope, cardiac arrest	Cephs, FQs, PCNs,
II (Delayed)	Antibody-mediated hypersensitivity	Antibiotic binds to WBC, RBC, or platelet and acts as antigen leading to antibody (usually IgG or complement) mediated cell destruction	7–14 d	Hemolytic anemia, thrombocytopenia, neutropenia	Cephs, PCNs, SMX/TMP
III (Delayed)	Immune complex mediated hypersensitivity	Antibiotic and IgG/IgM bind to form immune complex activate complement	7–14 d	Serum sickness *, vasculitis	Cephs (esp cefaclor), cipro, PCNs, SMX/TMP
IV (Delayed)	Delayed type hypersensitivity	Antigen specific T-cell activation			
	IVa	Monocytic inflammation (Th1 and IFN-γ)	10–15 d	Allergic contact dermatitis	Topical neomycin, bacitracin, polymyxin
	IVb	Th2-mediated eosinophilic inflammation	2–8 wk (for DRESS)	DRESS	PCNs, Cephs, Dapsone, MinocyclineSMX/TMP, Vanco
	IVc	CD8 T cell-mediated cytotoxicity	4–28 d	SJS, TEN	FQs, Nevirapine, PCNs, SMX/TMP
	IVd	T-cell-mediated neutrophilic inflammation	24–48 h	AGEP	Ampicillin, Antifungals, FQs, SMX/TMP

AGEP: Acute generalized exanthematous pustulosis. Cephs: cephalosporins. d: days. DRESS: drug rash with eosinophilia and systemic symptoms. FQ: flouroquinolones. h: hours. N/V: nausea/vomiting. PCNs: penicillins. RBC: red blood cell. WBC: white blood cell. SJS: Steven Johnson Syndrome. SMX/TMP: sulfamethoxazole/trimethoprim. SOB: shortness of breath. TEN: Toxic epidermal necrolysis. Vanco: vancomycin. wk: week. * Antibiotics in this group *mimic* serum sickness and cause a serum sickness-like reaction that is very similar based on symptoms but does not involve the production of immune mediated complexes.

**Table 2:** Chemical mediators of anaphylaxis and their effects on organ involvement [9,10,11,12,13].

Organ System	Symptoms	Main Mediators
GI	N/V, diarrhea, abdominal pain	Histamine
Skin	Flushing, urticaria, itching	Histamine PAF CysLTs
Respiratory	Dyspnea, bronchoconstriction, stridor, wheezing, cough, angioedema	Histamine Tryptase PAF CysLTs
CV	Hypotension, syncope, increased vascular permeability, vasodilatation	Histamine Tryptase PAF

CV: cardiovascular. CysLTs: cysteinyl leukotrienes. GI: gastrointestinal. PAF: platelet activating factor.

**Table 3:** Diagnosis of anaphylaxis [21].

Anaphylaxis Is Highly Likely If at Least One of the Following Three Criteria Is/Are Met:
1. Acute onset of symptoms with involvement of skin, mucosal tissue, or both (e.g., urticarial, pruritus or flushing, swollen lips–tongue–uvula) AND at least one of the following: Respiratory compromise (e.g., dyspnea, wheezing, bronchospasm, stridor, reduced PEF, hypoxemia) Reduced BP or symptoms of end-organ dysfunction (e.g., hypotonia, syncope, incontinence)
2. Two or more of the following that occur rapidly after exposure to a likely allergen (minutes to several hours): Involvement of the skin–mucosal tissue Respiratory compromise Reduced BP or associated symptoms Persistent GI symptoms (e.g., crampy abdominal pain, vomiting)
3. Reduced BP after exposure to known allergen for that patient (SBP <90 mmHg or >30% reduction from baseline)

BP: Blood pressure. PEF: peak expiratory flow. SBP: systolic blood pressure.

**Table 4:** Clinical criteria to assess likelihood of drug-induced thrombocytopenia [36].

Criteria	Description
1	Therapy with the suspected drug preceded thrombocytopenia; Recovery was complete and sustained after drug was discontinued
2	Other drugs administered prior to thrombocytopenia were continued or reintroduced after discontinuation of the suspected drug
3	Other causes of thrombocytopenia were excluded
4	Re-exposure to the suspected drug resulted in recurrent thrombocytopenia
*Levels of Evidence*	
Definite	All criteria met
Probable	Criteria 1–3 met
Likely	Criterion 1 met
Unlikely	Criterion 1 not met

**Table 5:** Steven–Johnson Syndrome (SJS) and Toxic Epidermal Necrolysis (TEN) Classification.

Name	% of BSA with Epidermal Detachment
SJS	<10
SJS/TEN overlap	10–30
TEN	>30

BSA: body surface area.
